# Established and emerging use of sodium-glucose cotransporter-2 inhibitors in pediatric population with type 2 diabetes: a case report and literature review

**DOI:** 10.3389/fendo.2025.1629488

**Published:** 2025-09-10

**Authors:** Valeria Castorani, Eleonora Chiarle, Silvia Savastio, Erica Pozzi, Cristina Partenope, Ciro Pignatiello, Sotirios Dimarakis, Monica Demichelis, Federico Medina, Ivana Rabbone

**Affiliations:** Division of Pediatrics, Department of Health Sciences, University of Piemonte Orientale, Novara, Italy

**Keywords:** type 2 diabetes, children, SGLT2 inhibitors, empagliflozin, hyperglycemia

## Abstract

The incidence and prevalence of type 2 diabetes (T2D) are increasing worldwide, affecting both adults and a growing number of children and adolescents. Notably, youth-onset T2D differs in its pathogenesis from later-onset T2D and progresses more rapidly. It is associated with a higher incidence of complications, and these occur earlier. Over the past few decades, numerous therapeutic agents from various drug classes have been approved for use in adults with T2D. In contrast, there is a scarcity of approved treatments for T2D in children. Sodium-glucose cotransporter-2 (SGLT2) inhibitors represent one of the newest classes of oral hypoglycemic agents, which lower blood glucose concentrations by increasing urinary glucose excretion. They have significantly influenced the management of T2D in adults. Promising results have also been reported in the pediatric population. In particular, empagliflozin, a potent and highly selective SGLT2 inhibitor, is an effective and generally well-tolerated anti-hyperglycemic agent widely approved for the treatment of adults with T2D. It has been recently approved for the management of T2D in children aged 10 years and older, in combination with diet and exercise. Here, we report a case of empagliflozin use in a 14-year-old girl with poorly controlled T2D. Furthermore, we review existing data on the use of SGLT2 inhibitors in the pediatric population.

## 1 Introduction

Type 2 diabetes (T2D) has become a significant global health concern, affecting both adults and an increasing number of children and adolescents, with relevant implications for public health systems.

According to the International Diabetes Federation (IDF), more than 537 million adults aged 20 – 79 were affected by T2D globally in 2021, with an estimated global healthcare expenditure of USD 966 billion, representing a staggering 316% increase over the past 15 years. Projections indicate that this number could rise to 783 million by 2045 if current trends continue (1–3).

In the pediatric population, the incidence of T2D has also shown a disturbing increase. Historically, T2D was considered rare in children and adolescents but over the past two decades the prevalence has surged, especially in populations with high rates of obesity. The SEARCH for Diabetes in Youth study revealed an annual increase of 4.8% in the incidence of T2D among U.S. children from 2002 to 2012 (4). Additionally, some authors highlighted that the prevalence of youth-onset T2D during adolescence (typically between the ages of 10 and 18), has risen 2 to 3 times compared to 30 years ago, with an estimated 41.600 new cases worldwide in 2021, especially in China, India, and the United States (5).

The mechanisms behind the rise of T2D in both adults and children are multifactorial. Genetic predisposition, environmental factors and the increasing prevalence of obesity have all been implicated. However, the pathophysiology of T2D in youth differs from that in adults with a more rapid decline in pancreatic β-cell function (about 20 - 35% per year in youth versus about 7 - 11% per year in adults), suggesting that T2D in youth is a more aggressive and severe condition (6, 7). In particular, obesity and insulin resistance play a relevant role in the pediatric population, where rapid weight gain during puberty exacerbates metabolic dysfunction. Peripheral insulin resistance is a key feature that occurs early in the disease course, and initially is compensated by increased insulin secretion reflected in hyperinsulinemia. Sustained hyperglycemia over time results in beta cell exhaustion and declining insulin secretion (glucose toxicity). This places children at greater risk of early onset complications such as nephropathy, retinopathy and cardiovascular disease, which are associated with long-term diabetes in adults.

**Figure 1:**
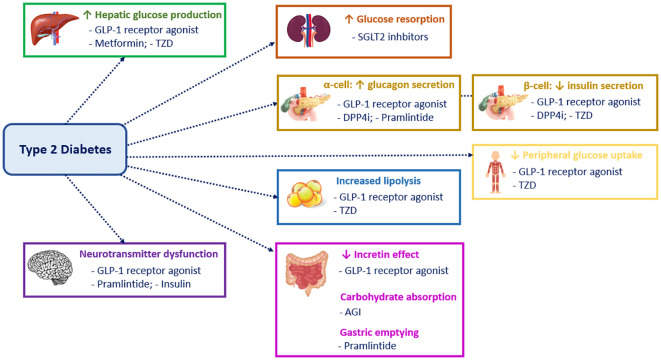
Mechanism of action of the different classes of type 2 diabetes drugs. GLP1, Glucagon-like peptide 1; TZD, Thiazolidinediones; SGLT2, Sodium-glucose cotransporter-2; DPP4i, Dipeptidyl peptidase-4 inhibitors; AGI, Alpha-glucosidase inhibitors.

**Figure 1** displays different targets and mechanisms of action of the many therapeutic agents and drug classes that have been approved for use in adults with T2D (8, 9). By contrast, there is a paucity of treatments for T2D in youth. Up to 2019, metformin and insulin were the only approved treatment options in children and adolescents with T2D. However, an increasing number of clinical trials of agents in youth-onset T2D have been completed or are nearing completion (7).

Sodium-glucose cotransporter-2 (SGLT2) inhibitors are one of the newest classes of oral hypoglycemic agents which lower blood glucose concentrations by increasing urinary glucose excretion. They have significantly influenced the management of T2D in adults; some of these medications, like dapagliflozin and empagliflozin, have recently been approved for the treatment of T2D in children and adolescents with promising results. As further evidence we present a clinical case of a 14-year-old female patient with T2D, who was successfully treated with empagliflozin therapy.

## 2 Case presentation

A 14-year-old female patient presented to our center with poorly controlled T2D, requiring multiple daily insulin injections (MDI) since diagnosis. Her previous medical history was uneventful; she was born full term after a pregnancy complicated by gestational diabetes. She was diagnosed with T2D at the age of 10, when she presented with episodes of hyperglycemia accompanied by sweating, polyuria, polydipsia, and fatigue. Blood tests showed negative type 1 diabetes autoantibodies and negative genetic testing for monogenic diabetes. An abdominal ultrasound showed a mildly thinned and heterogeneous-appearing pancreas; fecal elastase, lipase and pancreatic amylase were normal. Her family history was notable for T2D in maternal grandparents, and mother was affected by thyroid cancer.

At her first visit to our center, the patient appeared to be in good general condition. She presented with obesity (BMI 30 kg/m²). Her examination shows the presence of acanthosis nigricans on her neck and armpits. Her daily MDI therapy consisted of 9 units (U) of insulin glargine and 12 U of insulin lispro before meals (0.25 U/kg/day); her mean glycate hemoglobin (HbA1c) was 8.5%. Given the results of her previous tests, we decided to slightly reduce the total daily insulin dose and to start metformin therapy. Given the initial improvement, we gradually stopped insulin therapy and increased metformin dose to 2 g per day, together with a hypocaloric diet after consultations with dietitians and 10 hours per week of physical activity. After 12 months, this therapy resulted in weight improvement, with a weight loss of 10 kg and a BMI reduction from 30 to 26.4 kg/m², and better glycemic control, as demonstrated by a reduction in HbA1c levels (7.4%).

However, one year later, despite these initial improvements, her glycemic control remained suboptimal, with a time in range (TIR) of 61%. Considering the patient’s persistent hyperglycemia and the maternal history of thyroid cancer, which made Glucagon-Like Peptide-1 receptor agonists (GLP1-RA) less suitable, empagliflozin therapy was initiated at a dose of 10 mg daily, while maintaining metformin therapy. After six months of empagliflozin treatment, the patient’s glycemic control significantly improved: HbA1c levels decreased from 7.4% to 6.2% and TIR increased from 61% to 91%. She maintained a stable weight of 75 kg with a BMI of 26.2 kg/m².

No adverse events, such as diabetic ketoacidosis (DKA) or genital infections, were reported during the follow-up period. **Table 1** shows her progresses over time.

**Table 1:** Anthropometric measurements and data on glycemic control and treatments of the patient at first visit and during follow-up.

Parameters	1st visit (20/03/2023)	2nd visit (01/12/2023)	3rd visit (06/02/2024) START empagliflozin	4th visit (14/06/2024)	Last visit (28/03/2025)
Age (years)	13.3	14	14.2	14.6	15.3
Height (cm)	168.5	169	169	169.4	170
Weight (kg)	85.3	77.5	75.4	75.2	74.8
BMI (kg/m²)	30	27.1	26.4	26.2	25.8
Treatment	Glargine 9 U/d + Lispro 12 U/d	Metformin 1500 mg/d	Metformin 2000 mg/d	Metformin 2000 mg/d + Empaglifozin 10 mg	Metformin 2000 mg/d + Empaglifozin 10 mg
HbA1c (%)	8.5	7.7	7.4	6.4	6.5
TIR (%)	63	68	62	91	85
Blood pressure (mmHg)	114/58	96/62	100/72	100/56	110/64
Liver function tests (U/L)			AST 13 ALT 8		AST 15 ALT 9
Lipid profile (mg/dL)		Cholesterol total 132 LDL 79 HDL 44 TG 43			Cholesterol total 135 LDL 87 HDL 42 TG 28
Renal function test (mg/dl)			Creatinine 0.65 ACR 1.5		Creatinine 0.75 ACR 3.3

BMI, Body mass index; HbA1c, glycated hemoglobin; TIR, Time in range; AST, aspartate aminotransferase; ALT, alanine aminotransferase; LDL, low-density lipoproteins; HDL, high-density lipoproteins; TG, triglycerides; ACR, urine albumin to creatinine ratio.

## 3 Discussion

This study describes the innovative and effective use of empagliflozin in a pediatric patient with T2D. The American Diabetes Association (ADA) and the European Association for the Study of Diabetes (EASD) recommend SGLT2 inhibitors as a cornerstone of T2D treatment in adults (10). Currently, SGLT2 inhibitors such as dapagliflozin and empagliflozin are the only additional oral agents to have received regulatory approval from the European Medicines Agency (EMA) for use in children aged 10 years or older (11), addressing a previous scarcity of treatment options beyond metformin and insulin in this population. Pharmacodynamic and pharmacokinetic studies have elucidated that approximately 180g of glucose per day is excreted through glomerular filtration into the primary urine. This glucose is then almost completely reabsorbed by SGLT1 and SGLT2 in the proximal tubule via sodium-glucose cotransport across apical cell membranes (12, 13). In humans, six different SGLT isoforms have been identified (14). SGLT2 is highly expressed in the early segments (S1-S2) of the proximal renal tubule and reabsorbs approximately 90% of filtered glucose, while SGLT1 normally reabsorbs the remaining glucose in the S3 segment. In patients with hyperglycemia, the maximum reabsorption capacity is amplified; this amplification, coupled with an increased concentration of systemic glucose, results in persistent hyperglycemia and glucotoxicity, leading to β-cell dysfunction (15).

**Figure 2:**
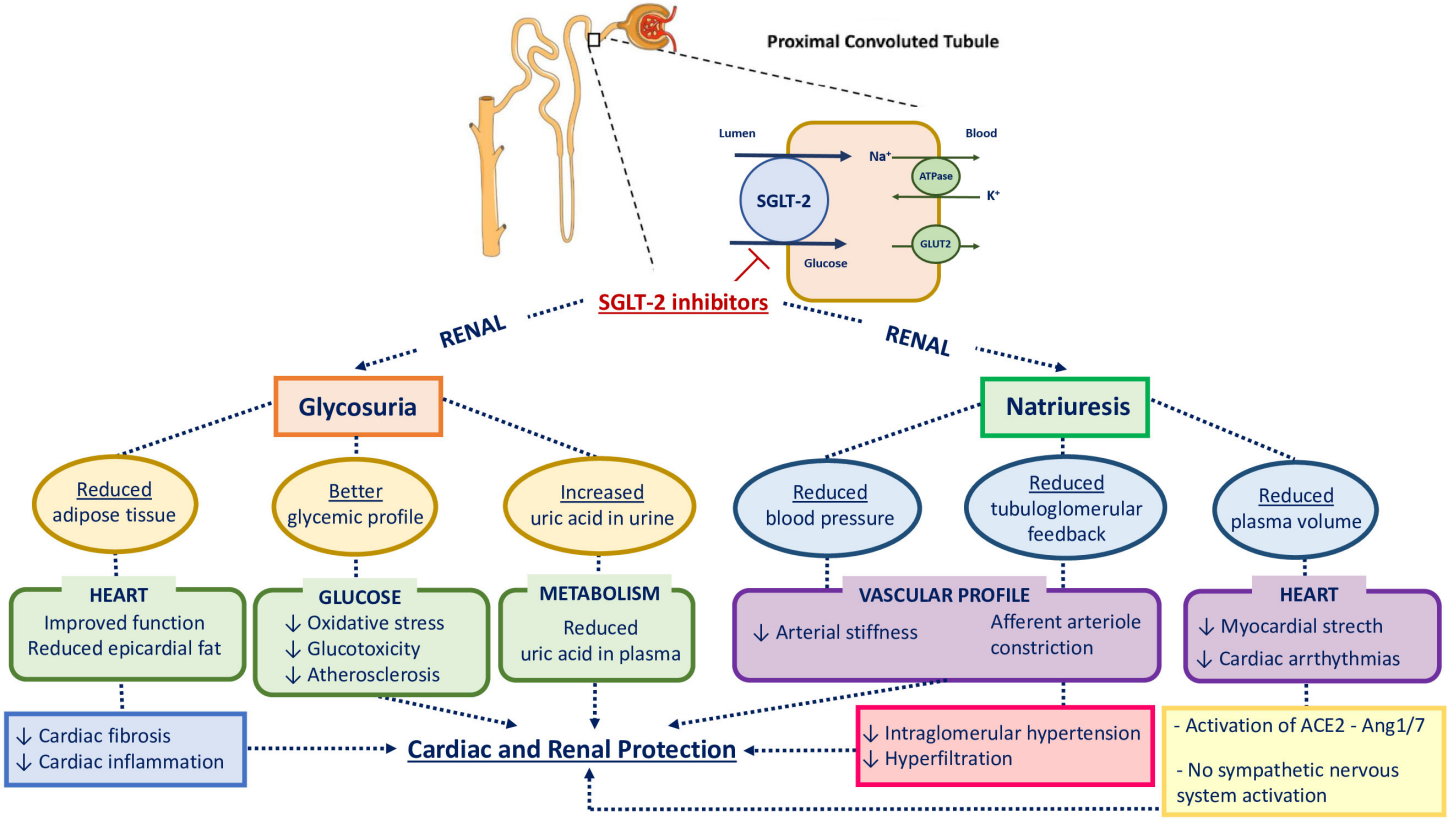
Physiological mechanisms involved in cardiovascular and renal protection with SGLT2 inhibition. HbA1c, glycated hemoglobin; SGLT2, sodium-glucose cotransporter-2; GLUT2, glucose transporter type 2; ACE2, angiotensin-converting enzyme 2; Ang 1 - 7, Angiotensin 1 - 7.

SGLT2 inhibitors reduce the maximum reabsorption of glucose, thereby increasing urinary glucose excretion and lowering blood glucose concentration in an insulin-independent manner, as depicted in **Figure 2**, according to previous studies (16–18). Consequently, they improve β-cell function by alleviating glucotoxicity and potentially reducing β-cell workload (19). Furthermore, the excretion of glucose leads to energy loss from the body, promoting weight loss; conversely, glucagon secretion increases after the administration of SGLT2 inhibitors, thus contributing to the promotion of lipolysis and the reduction of liver fat and visceral adiposity (20). The improvement of overweight and obesity promotes a reduction in insulin resistance and other metabolic parameters, such as blood pressure, lipid profile, and uric acid levels (17, 20).

We herein describe the progresses in treatment of pediatric T2D in a real-life setting. Metformin and insulin were the established treatments for T2D in youth until the recent approval by the Food and Drug Administration (FDA) of two SGLT2 inhibitors (dapagliflozin and empagliflozin) for children and adolescents above 10 years of age with T2D, for use in combination with diet and exercise. This approval marked a significant advancement in the therapeutic landscape for this vulnerable population. **Table 2** lists previous studies about the effects of these treatments in the pediatric population.

**Table 2:** Summary of the main pediatric studies on the use of SGLT2 inhibitors.

Reference	Drug	Study design	Sample size	Efficacy	Adverse Events
Tamborlane WV et al. (21) 2020	Dapagliflozin vs Placebo	Placebo-controlled double-blind randomized phase 3	72	Reduction in HbA1c (-0.25% Dapagliflozin vs +0.50% Placebo)	Headache (10.3% vs 9.1%) Nasopharyngitis (10.3% vs 0%) Vitamin D deficiency (10.3% vs 3%) Urinary tract infections (5.1% vs 3.0%) ≥1 Hypoglycemia (28.2% vs 18.2%) No episodes of DKA
Shehadeh N et al. (22) 2023	Dapagliflozin vs Saxagliptin vs Placebo	Placebo-controlled double blind parallel group randomized phase 3	256	Reduction in HbA1c (-1.03% Dapagliflozin vs placebo; - 0.44%Saxagliptin vs placebo)	Headache (14.8% vs 3% vs 5.3%) Severe hypoglycemia (4.9% vs 4.5% vs 7.9%) No episodes of DKA
Laffel LM et al. (23) 2018	Empagliflozin (5 mg vs 10 mg vs 25 mg)	Single dose phase I open label randomized parallel group	27	After a single dose of empagliflozin, adults and young with T2D had similar exposure- response relationships after adjusting for significant covariates	1 event of mild intensity ‘dehydration’ (empagliflozin 10 mg) 1 case of hypoglycemia (plasma glucose of 3.8 mmol/l at 32 h after empagliflozin administration) No episodes of DKA
Laffel LM et al. (31) 2023	Empagliflozin 10mg vs oral Linagliptin 5mg vs Placebo	Double-blind placebo-controlled	158	Reduction in HbA1c (-0.84% Empagliflozin; - 0.34% Linagliptin)	Hypoglycemia (19.3% Empagliflozin vs 22.1% Linagliptin) Gastrointestinal symptoms (25.5% Empagliflozin vs 27.9% Linagliptin) Urinary tract infections (7.2% Empagliflozin 1.5% Linagliptin) DKA (0% Empagliflozin 2.9% Linagliptin)

SGLT2, sodium-glucose cotransporter-2; HbA1c, glycated hemoglobin; T2D, type 2 diabetes; DKA, diabetic ketoacidosis.

Specifically, dapagliflozin was the second oral treatment following metformin to be approved by the EMA in children 10 years of age or older, following a phase 3 trial (11, 21). This multicenter, randomized, parallel-group study evaluated the efficacy and safety of dapagliflozin versus placebo in young people aged 10 – 24 years with T2D (HbA1c 6.5%-11%). Seventy-two participants were randomized to dapagliflozin 10 mg or placebo during a 24-week double-blind period, followed by a 28-week open-label safety extension where all participants received dapagliflozin.

After 24 weeks, there was a statistically significant decrease in HbA1c with dapagliflozin compared to placebo. Over the entire 52 weeks of the study, AEs occurred in 74.4% of participants randomized to dapagliflozin, the most common were headache, nasopharyngitis, and vitamin D deficiency. No episodes of DKA were observed, and the only AE that led to treatment discontinuation was a genital infection. Hypoglycemia occurred in one-third of participants with dapagliflozin (almost all of whom were also receiving insulin), mainly asymptomatically; no hypoglycemia events led to discontinuation of treatment. There was no notable effect of treatment on body weight, BMI, or blood pressure (21).

More recently, the T2NOW trial compared dapagliflozin and saxagliptin (a dipeptidyl peptidase-4 inhibitor (DPP - 4i)) separately versus a single placebo group. This was a 52-week trial with a 26-week extension among children (10 – 17 years of age) with uncontrolled T2D (HbA1c 6.5 - 10.5%) receiving metformin, insulin, or both. Participants were randomly assigned to 5 mg of dapagliflozin, 2.5 mg of saxagliptin, or placebo. With dapagliflozin, a significant decrease in HbA1c and fasting plasma glucose was observed early in treatment and sustained over 52 weeks, statistically significant versus placebo. The most common AE was headache, followed by hypoglycemia, which occurred in almost one-third of patients; most events were mild, and none was considered serious or resulted in discontinuation. No new safety signals were observed (22). Compared with the previous phase 3 trial of dapagliflozin (21), in which most patients were either White or Black/African American, this trial was more representative of the ethnically diverse population affected by T2D globally (22), potentially enhancing the generalizability of its findings.

The second SGLT2 inhibitor, empagliflozin, was tested in a randomized trial assessing its pharmacokinetic and pharmacodynamic profile and identifying the appropriate doses in young people with T2D aged 10 to 17 years. The most important finding of this study was that the pharmacokinetic characteristics of the 10 and 25 mg doses of empagliflozin approved for use in adults were generally similar in young people with T2D. As observed in adult patients, the pharmacodynamic actions of empagliflozin showed dose-dependent increases in urinary glucose excretion and corresponding decreases in fasting plasma glucose and mean daily glucose levels. Protocol-specified AEs of specialinterest (hepatic injury, decreased renal function, and DKA) were not reported. No meaningful changes in systolic and diastolic blood pressure were observed (23), as confirmed in our patient. This contrasts with studies in adults with T2D, that described the cardiovascular benefits of this drug in comparison to GLP - 1 Ras and placebo (24, 25), although the reason for this difference remains unclear (26).

Our patient showed normal renal function at baseline and during treatment with empagliflozin. SGLT2 inhibitors have also consistently shown renoprotective effects in adults (27–30), but these effects have not been explored in the pediatric population.

The most recent randomized, double-blind, parallel-group, Phase 3 DINAMO trial assessed the efficacy and safety of empagliflozin versus placebo on glycemic control in children and adolescents with T2D who had been previously treated with metformin or insulin. One hundred fifty-eight participants aged 10 – 17 years (HbA1c 6.5 - 10.5%) were randomly assigned to oral empagliflozin 10 mg, oral linagliptin 5 mg (DPP - 4i), or placebo. Participants in the empagliflozin group who did not achieve an HbA1c below 7% by week 12 underwent a second randomization, either remaining on 10 mg or increasing to 25 mg. Participants in the placebo group were randomly reassigned at week 26 to linagliptin 5 mg or one of the empagliflozin doses. The study demonstrated that empagliflozin provided clinically relevant placebo-corrected reductions in HbA1c, whereas linagliptin did not. The safety profile of empagliflozin was comparable to that observed in adult patients: hypoglycemia was the most frequently reported adverse event, with higher rates for those on active drug treatment compared with placebo. No severe hypoglycemia nor DKA were reported. The occurrence of urinary tract infections were slightly higher in the empagliflozin group than in the placebo group (31). Our patient did not show any AE over more than one year of treatment with empagliflozin.

## 4 Conclusion

T2D is becoming increasingly prevalent not only in adults but also in the pediatric population; it represents one of the most significant risk factors for both microvascular and macrovascular complications. Recent advances have been made in the treatment of T2D. In particular, SGLT2 inhibitors have revolutionized the management of diabetes, offering not only effective glycemic control but also significant cardiovascular and renal benefits demonstrated in adult studies. While numerous studies have evaluated their efficacy and safety in adults, fewer studies have focused on the pediatric population. The recent approval of empagliflozin for children and adolescents with T2D marks a crucial step forward, with initial studies reporting promising results. In the clinical case we presented, the use of empagliflozin enabled us to achieve excellent glycemic control in our 14-year-old patient, a result that had never been reached before with metformin therapy and lifestyle intervention, highlighting its potential in managing this challenging condition. The reported data are consistent with existing literature and further support the applicability of SGLT2 inhibitors in real-world clinical practice. Youth-onset T2D presents a significant clinical challenge due to its rapid progression and increased risk of early complications, underscoring the need for novel and effective therapeutic strategies. This new therapeutic approach with SGLT2 inhibitors could potentially change the course of the disease in young individuals. Further studies are warranted to establish the long-term safety and efficacy of SGLT2 inhibitors in the pediatric age group and to optimize their use in this specific population.

## Data Availability

The raw data supporting the conclusions of this article will be made available by the authors, without undue reservation.
